# Well-Child Visits and Immunization Encounters in South Carolina Medicaid: A 3-Year Retrospective Comparison Between Rural and Urban Children with a History of Neonatal Opioid Withdrawal Syndrome, 2006–2014

**DOI:** 10.3390/healthcare13131539

**Published:** 2025-06-27

**Authors:** Farah Tahsin, Leah Holcomb, Elizabeth Charron, Lori Dickes, Rachel Mayo, Windsor Sherrill, Jennifer Hudson, Julie Bedi

**Affiliations:** 1 Department of Political Science, 2023 Barre Hall, Clemson University, Clemson, SC 29634, USA; lorid@clemson.edu; 2 Department of Public Health Sciences, 503 Edwards Hall, Clemson University, Clemson, SC 29634, USA; holcomle@musc.edu (L.H.); rmayo@clemson.edu (R.M.); wsherri@clemson.edu (W.S.); 3 Department of Internal Medicine, University of Utah School of Medicine, 295 Chipeta Way, Salt Lake City, UT 84132, USA; elizabeth-charron@ouhsc.edu; 4 Program for Addiction Research, Clinical Care, Knowledge and Advocacy, Division of Epidemiology, University of Utah School of Medicine, 383 S Colorow Dr, Salt Lake City, UT 84103, USA; 5 Newborn Services, Prisma Health Upstate, 701 Grove Road, Greenville, SC 29605, USA; jennifer.hudson2@prismahealth.org; 6 Scale Strategic Solutions, Cincinnati, OH 45206, USA; jsb@scalestrategicsolutions.com

**Keywords:** opioids, neonatal opioid withdrawal syndrome, well-child visits, immunization, medicaid, policy

## Abstract

**Background/Objectives:** This retrospective cohort study compared well-child visits (WCVs) and immunization encounters from birth to age three among rural and urban South Carolina (SC) Medicaid-enrolled children with neonatal opioid withdrawal syndrome (NOWS). **Methods:** We applied logistic and Poisson regression models to examine associations between rural status and the number of WCVs, WCV adherence, and immunization encounters. **Results**: The sample included 833 urban and 161 rural children with NOWS born between 2006 and 2014. Significant differences existed between groups in the number of WCVs and immunization encounters each year from birth to age three (*p* = < 0.01 for all the comparisons). After covariate adjustment, rural compared to urban status was associated with decreased WCVs from birth to 11 months (incidence rate ratio (IRR): 0.85; 95% CI: 0.77–0.93) and 12 to 23 months (IRR: 0.80; 95% CI: 0.69–0.93). Rural status was not significantly associated with decreased WCVs from 24 to 35 months (IRR: 0.81; 95% CI: 0.63–1.03). Rural compared to urban status was associated with a 34% lower odds of WCV adherence from 12 to 23 months (odds ratio (OR): 0.66; 95% CI: 0.44–0.99). Furthermore, rural compared to urban status was associated with decreased immunization encounters from birth to 11 months (IRR: 0.60; 95% CI: 0.52–0.69), 12 to 23 months (IRR: 0.61; 95% CI: 0.50–0.71), and 24 to 35 months (IRR: 0.55; 95% CI: 0.40–0.76). **Conclusions:** Rurality was associated with decreased WCVs and immunization encounters among children with a history of NOWS residing in SC. Policy interventions, including telehealth services and expanded Medicaid access, could improve WCV and immunization rates among these children.

## 1. Introduction

Rates of maternal opioid use disorder (OUD) and neonatal opioid withdrawal syndrome (NOWS) are rising in the United States (U.S.), particularly among rural populations [1,2]. Between 2003 and 2013, the rate of NOWS in rural communities increased from approximately 1 to 7 per 1000 births, nearly 80% higher than the rate of increase in urban areas [3]. In 2016, the rate of NOWS among infants born to women residing in rural areas was 10.6 per 1000 [4]. Evidence suggests that NOWS may be associated with ongoing health conditions, such as behavioral problems, that require early and sustained intervention from pediatric healthcare providers [5,6]. Well-child visits (WCVs) offer a critical opportunity for children with a history of NOWS to receive developmental and socioemotional screening as well as essential preventive care, such as immunizations [7,8].

The American Academy of Pediatrics (AAP) recommends that children have six WCVs during the first year of life, at 1, 2, 4, 6, 9, and 12 months [9]. Regular WCV attendance has been linked to decreased hospitalizations and allows for timely immunization updates [10]. Immunization encounters are typically scheduled at 2, 4, 6, 12, and between 15 and 18 months of age and during the influenza season [11]. The CDC recommends early childhood immunizations against hepatitis A and B, rotavirus, diphtheria, tetanus, acellular pertussis, influenza, measles, mumps, rubella, and varicella [11]. Children with NOWS are recommended to follow the standard immunization schedule; however, adherence to this schedule in this population is not well understood. Examining WCVs and immunization encounters in the NOWS population is, therefore, vital, as there is limited research measuring long-term effects of NOWS on childhood health and healthcare utilization [12,13].

Given the higher prevalence of opioid use, poverty, and limited healthcare access in rural areas, it is important to examine geographic disparities in WCV adherence and immunization encounters among children with NOWS [14]. Rural children are more likely to live in poverty, rely on public insurance, and experience worse health outcomes [15]. Rural areas also face shortages of primary care providers and limited access to ancillary services such as physical, occupational, and speech therapy [16]. Access to immunization encounters is also limited in rural areas, where fewer providers, longer travel times, and fewer care centers can delay or prevent receipt of care [17,18]. As a result, children with NOWS living in rural areas may face healthcare access barriers not experienced by their urban counterparts [1]. National data show that children with NOWS have lower WCV adherence rates than non-exposed children, even when geographic factors are not considered [8]. However, rural/urban differences in WCV use among children with NOWS have not been examined, despite known disparities in healthcare access. The objective of this study was to examine WCV adherence from birth to age three between rural and urban children with NOWS and, secondarily, to examine differences in immunization encounters between groups.

## 2. Materials and Methods

### 2.1. Data Source and Analytic Sample

We conducted a retrospective cohort study using linked South Carolina (SC), U.S. Medicaid claims and birth certificate data to identify a population-based sample of children diagnosed with NOWS at birth. The cohort was constructed from live births occurring between 2006 and 2014 to Medicaid-enrolled mothers. Birth certificate records were linked to Medicaid data using a unique identifier provided by the South Carolina Office of Revenue and Fiscal Affairs (SCRFA), Columbia, SC, U.S. Medicaid data include enrollment information, inpatient and outpatient medical claims (diagnoses and procedures), and pharmacy claims. In 2016, Medicaid was the primary payer for 83% of hospital charges related to NOWS in the U.S. [4], making it a suitable data source for capturing this population. Children were followed for 3 years after their birth hospitalization, resulting in a study period spanning 2006 to 2017.

Infants with NOWS were identified using ICD-9-CM code 779.5 listed in 1 of up to 14 discharge diagnosis fields during the birth hospitalization. This code has demonstrated high sensitivity and positive predictive value for identifying clinically diagnosed NOWS in administrative datasets [12,13]. To ensure adequate follow-up, we limited the sample to children with continuous Medicaid enrollment for at least 9 out of 12 months per year through age 3. We excluded infants with clinical conditions consistent with iatrogenic withdrawal, given that these children often have longer hospital stays and may follow a different care trajectory [19,20]. Iatrogenic NOWS was identified using a previously published claims-based algorithm [21,22] and included diagnoses of intraventricular hemorrhage (n = 25), periventricular leukomalacia (n = 2), necrotizing enterocolitis (n = 6), spontaneous intestinal perforation (n = 0), and bronchopulmonary dysplasia (n = 19). We also excluded infants with low birth weight (<2500 g; n = 54) to minimize confounding by complications related to prematurity.

The final analytic sample included 994 children with NOWS, including 161 classified as rural and 833 as urban based on maternal county of residence at birth (Supplemental Material, Figure S1). Study participants represented 44 of South Carolina’s 46 counties; only two counties had no infants diagnosed with NOWS during the study period. This study was deemed exempt by the Clemson University Institutional Review Board.

### 2.2. Outcomes

We examined WCVs, WCV adherence, and immunization encounters from birth to age 3. Because birthdates are not available in SC Medicaid claims, we could not determine the exact age of the participants at the time of each healthcare encounter. Instead, the number of months the encounter took place past the birth month was used as a proxy for age. We approximated the first year of life as 0–11 months of age, the second year of life as 12–23 months, and the third year of life as 24–35 months. To derive outcomes, we used Current Procedural Terminology (CPT) codes for pediatric preventive care recommended by the AAP [23]. (Supplement Table S1). Consistent with the AAP guidelines for WCVs and prior research [7,8], we defined WCV adherence in year 1 as a completed visit by month 1 and four additional visits by 11 months; adherence in years 2 and 3 was defined as at least two completed visits between 12 and 23 months and at least one completed visit between 24 and 35 months, respectively.

Given that WCVs are an opportunity to update and complete a child’s immunizations [9], we also examined the number of outpatient visits containing at least one immunization (hereafter referred to as ‘immunization encounter’). Immunization encounters were captured using CPT codes (Table S1). Because many claims used generic CPT codes, we could identify how many encounters included one or more immunizations, but not which vaccines were administered. Per the Centers for Disease Control and Prevention’s recommended child and adolescent immunization schedule [11], a child would not receive an immunization at every WCV. Nevertheless, our measure provides insight into children’s receipt of immunization services during these formative years.

### 2.3. Rural Status

We defined rural and urban residence using the 2013 Rural/Urban Continuum Codes (RUCC) developed by the U.S. Department of Agriculture. RUCC classifies all U.S. counties into nine categories based on population size and proximity to metropolitan areas. Categories 1–3 represent metropolitan (urban) counties, while categories 4–9 represent nonmetropolitan (rural) counties [24,25]. We applied RUCC at the county level using the maternal county of residence at the time of delivery, classifying the children as rural if their county was assigned an RUCC of 4–9 and urban if assigned a code of 1–3. This classification method is widely used in rural health research and is recognized by the USDA as the most common approach for distinguishing rural and urban areas [26].

### 2.4. Covariates

Covariates included the demographic and clinical characteristics of the mothers and children and were based on clinical opinion and a review of the literature. Child sex, race/ethnicity, birth year, and maternal age were obtained from Medicaid data. Child gestational age, birth weight, maternal education, number of previous live births, adequacy of prenatal care, and tobacco use during pregnancy were obtained from birth certificate data. The Kotelchuck Adequacy of Prenatal Care Utilization Index was used to classify prenatal care adequacy [27].

### 2.5. Statistical Analyses

We calculated descriptive statistics stratified by rural versus urban status. Between-group differences were examined using Student’s *t*-tests for continuous normally distributed variables, two-sample Wilcoxon rank-sum tests for continuous skewed variables, and χ^2^ tests for categorical variables. The results of descriptive analyses were expressed as mean (standard deviation, SD), median (interquartile range, IQR), or n (%). We estimated Poisson regression models adjusted for all the covariates to examine associations between rural status and total count of WCVs and immunization encounters during the first 3 years of life for the primary analyses. To investigate the association of rural status with WCV adherence, we fit logistic regression models. All the models were adjusted for previously described covariates. For Poisson models, we assessed overdispersion and zero inflation and found no violations of model assumptions. We reported Poisson regression results as incidence rate (IRRs) and logistic regression results as odds ratios (ORs). Additionally, we reported 95% confidence intervals (CIs) for all the estimates.

Data were missing for several of our covariates, including gestational age (7.7%), birth weight (7.2%), previous live births (7.3%), maternal education (7.7%), adequacy of prenatal care (8.2%), and tobacco use in pregnancy (7.5%). Therefore, we performed multiple imputation using chained equations to estimate missing values for these variables [12]. To ensure that imputation did not affect our results, we performed a complete case analysis. A recent environmental scan found that while the most commonly used newborn diagnostic code for NOWS surveillance was 779.5, 25% of the surveyed jurisdictions also used 760.72 [28]. Accordingly, we conducted an additional sensitivity analysis including children identified with diagnosis codes 779.5 and 760.72 to check whether the results remained robust with two codes for NOWS identification. A two-sided significance level of 0.05 was used for all the statistical testing. We analyzed the data using SAS version 9.4 (SAS Institute, Cary, NC, USA) and Stata version 14.2 (StataCorp, College Station, TX, USA). We followed the STrengthening the Reporting of OBservational studies in Epidemiology (STROBE) reporting guidelines for cohort studies (Table S2) [29].

## 3. Results

Characteristics of the children and their mothers in our study are summarized in Table 1. There were no statistically significant differences in characteristics between the children from rural and urban areas. The median (IQR) gestational age was 38 weeks, and the mean (SD) birth weight was approximately 2950 g. The mothers’ median (IQR) age was 27 years for both groups. Across both groups, most mothers had inadequate prenatal care, and over one-third smoked during pregnancy.

**Figure 1:**
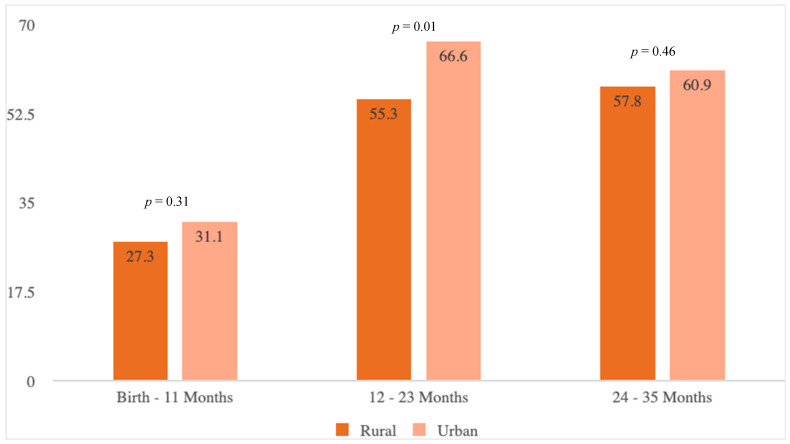
Well-child visit adherence during the first 3 years of life for South Carolina Medicaid-enrolled rural and urban children diagnosed with neonatal opioid withdrawal syndrome at birth, 2006–2014 (N = 994).

There were significant differences between children living in rural versus urban areas in the number of WCVs and immunization encounters during each year of life from birth to age three (*p* = < 0.01 for all comparisons) (Table 2). Between birth and 11 months, the mean (SD) number of WCVs for children living in rural areas was 3.4 (1.7) compared to 4.0 (2.0) for those living in urban areas. Children living in rural communities received an average of 1.6 (SD 1.6) immunization encounters between birth and 11 months, while those in urban areas completed an average of 2.6 (1.5) immunization encounters during the same period. Between 12 and 23 months, the children in rural areas had an average of 1.7 (1.1) WCVs and 1.4 (1.3) immunization encounters, while those in urban communities had an average of 2.1 (1.3) WCVs and 2.0 (1.2) immunization encounters. From 24 to 35 months, the mean (SD) number of WCVs and immunization encounters for the children living in rural communities was 0.6 (0.6) and 0.3 (0.6), respectively, compared to 0.7 (0.6) and 0.6 (0.7), respectively, for those in urban areas. The children in the rural group were significantly less likely to adhere to the AAP guidelines for WCV attendance from 12 to 23 months (55.3% vs. 66.6%; *p* = 0.01) when compared to the urban group (Figure 1).

After covariate adjustment, rural status was significantly associated with decreased WCVs from birth to 11 months and 12 to 23 months and decreased WCV adherence from 12 to 23 months (Table 3). There was a 15% and 20% respective decrease in the WCV rate between the rural and urban group from birth to 11 months (IRR: 0.85; 95% CI: 0.77–0.93) and 12 to 23 months (IRR: 0.80; 95% CI: 0.69–0.93). Compared to the children living in urban areas, those in rural areas had 34% lower odds of WCV adherence between 12 and 23 months (OR: 0.66; 95% CI: 0.44–0.99). Additionally, rural status was associated with decreased immunization encounters from birth to 11 months (IRR: 0.60; 95% CI: 0.52–0.69), 12to 23 months (IRR: 0.60; 95% CI: 0.50–0.71), and 24 to 35 months (IRR: 0.55; 95% CI: 0.40–0.76). Complete case and sensitivity analysis, including children identified with code 760.72, produced similar results to the main findings (Tables S3 and S4).

## 4. Discussion

This study compared well-child visit (WCV) utilization and immunization encounters from birth to age three among rural and urban children diagnosed with NOWS at birth. We found that the children residing in rural areas in SC had fewer WCVs and immunization encounters in the first 3 years of life than their urban counterparts, suggesting a significant gap in early childhood care for rural infants with NOWS. SC is a relatively rural state, with approximately 27% of individuals living in rural areas, indicating that many children may lack access to timely and consistent healthcare [30]. Given SC’s large rural population, examining geographic differences in WCV and immunization utilization helps identify critical gaps in care that may be targeted for improvement. Identifying specific areas of need can inform future policies and interventions to improve childhood healthcare access in rural areas.

Overall, neither rural nor urban groups met the recommended AAP guidelines for WCVs. Our results are consistent with previous studies that found children with NOWS have reduced WCVs during the first year of life compared to non-exposed children [8,31], and this disparity in healthcare access was more noticeable in rural children. As children age, a reduction in the number of WCVs follows the general trend among publicly insured children, which we also identified in our population of NOWS-diagnosed infants. Rurality only widened the gap in adherence to recommended care between the two groups. Although WCVs are the primary setting to regularly evaluate developmental markers and offer preventive and treatment services, many children, in general, are not receiving the baseline recommendations for WCVs [7]. While Medicaid expansion has been associated with improved access to care and reduced financial burden for low-income populations, evidence of its effect on pediatric preventive services remains mixed [32]. Children with NOWS often start life with unique challenges; fewer WCVs and on-time immunizations may exacerbate these challenges and contribute to adverse long-term health outcomes.

Rural health research highlights that rurality is, in part, associated with poorer health outcomes [31,33,34]. Due to provider shortages, transportation challenges, and other system-level barriers, rural families face more obstacles to adhering to the AAP-recommended WCV schedule [35]. Several ideas have been proposed to improve access to care for children living in rural areas. Telehealth services can mitigate healthcare access barriers such as transportation barriers, provider shortages, and a lack of insurance coverage that often trigger poor health outcomes and are disproportionately experienced in rural areas [36,37]. While telehealth can improve access to preventive care and developmental screening, its impact on immunization access is limited due to the need for in-person vaccine administration. Telehealth in conjunction with home visiting programs and expanded immunization clinics in rural areas will be necessary to improve immunization encounters in this population [38]. Home health nurses who visit NOWS babies and their families in the early days of life may also play an important role in supporting the coordination of early pediatric care, including WCVs and immunization for young children and their families in rural communities. Ultimately, additional research is needed to identify targeted and coordinated interventions that would increase access to WCVs and immunization encounters for NOWS infants living in rural areas.

To further reduce disparities between infants residing in rural and urban areas, increased insurance coverage through Medicaid and the state children’s health insurance programs (SCHIP) [39] could provide improvements in care access. SCHIP was established to ensure that children in low-income households, who are ineligible for Medicaid, had the availability of health insurance coverage [40]. It is crucial to consider the expansion of Medicaid coverage and increase SCHIP funding to ensure wider and longer access to care among children with NOWS [41]. To increase recommended immunization coverage, there is a need for improved coordination among state immunization programs [17,42]. Highly coordinated immunization programs can support timely immunization encounters for children with NOWS in rural areas by improving immunization opportunities and minimizing interruptions in insurance coverage [17]. Community pharmacy-based immunizations can be another potential strategy to decrease barriers to rural children’s access to immunizations [43].

Advanced practice providers (e.g., nurse practitioners and physician assistants) are essential for building trust with families by providing individualized, nonjudgmental care. Pharmacists also play a critical role in expanding immunization access by administering vaccines, promoting adherence, and serving as immunization advocates within the community [44]. Expanding the role of pharmacists in immunization delivery may help overcome provider shortages in rural areas and improve timely vaccine access for children with NOWS. Expanding pharmacists’ involvement in vaccine delivery may help address provider shortages in rural areas and improve timely access to immunizations for children with NOWS. Families affected by substance use and NOWS may be less likely to seek care due to stigma or fear of social service involvement [45]. Both advanced practice providers and pharmacists can help increase WCV and immunization rates in this population through targeted outreach and consistent care delivery. Pediatric practices in rural areas could also implement reminder and recall systems, along with case management strategies, to proactively schedule WCVs and immunization appointments for children with NOWS.

This study has several limitations related to the use of Medicaid claims data as the primary data source. These data are collected for billing rather than research purposes, which limits their granularity and the range of information available. Moreover, the data contains a high proportion of missing values. Although we used multiple imputation to address this, the extent of missingness remains a limitation and may introduce bias and reduce the precision of the estimates. Additionally, the findings may not be generalizable to children with NOWS without continuous Medicaid enrollment because children with gaps in coverage were excluded. Accordingly, the observed healthcare utilization patterns may not reflect those of children who disenroll or transition to other insurance within the first 3 years of life. We also did not have access to a centralized immunization registry to obtain a complete immunization history. These immunization encounters were also limited to outpatient visits and are not generalizable to immunizations given in other settings, like county health departments. The observed rural/urban gap in immunization encounters may also be partially influenced by data limitations. Specifically, immunizations administered in non-clinical settings such as county health departments or community events are not captured in Medicaid claims data. Therefore, rural families may disproportionately rely on such services, which could lead to an overstatement of the disparity. Other limitations include restricted dates that prevent an accurate calculation of child age, the risk of coding errors within claims data, and a lack of information on specific social or demographic characteristics that could enhance the analysis. For example, for geographic information, we can only access county-level data instead of more granular census tract data. Understanding the locational aspects of where these children receive medical services after birth could provide more insight into rural/urban care gaps. However, we applied multiple statistical methods to correct for some of these data challenges and sought as many relevant and justifiable data proxies that could serve to answer our core research questions.

We did not adjust for county-level variables such as household income, employment, educational attainment, or healthcare provider density, as these are likely mediators on the causal pathway between rural residence and WCV or immunization encounter utilization. Controlling for these variables would constitute overadjustment and could attenuate the total effect of rurality, which was our primary exposure of interest [46]. While structural factors at the county level may partially explain why rural children receive fewer WCVs and immunizations than urban children, adjusting for them would reduce model interpretability and obscure the overall relationship we sought to estimate.

## 5. Conclusions

In this population-based study of SC Medicaid enrollees, children with a history of NOWS who resided in rural areas received fewer WCVs and immunization encounters in the first 3 years of life compared with their urban counterparts. These disparities are concerning given the increased medical and developmental risks faced by children with NOWS. Preventive care in early childhood, including routine WCVs and timely immunizations, is important to promote healthy development and reduce long-term morbidity. Our study findings underscore the need for targeted interventions to improve access to pediatric preventive services in rural communities. Although Medicaid claims data may underestimate services delivered in non-clinical settings, particularly in rural areas, the rural/urban gap in care utilization remains evident. Strengthening early childhood care through expanded access to advanced practice providers, pharmacists, and community-based programs may help address these disparities and improve outcomes for high-risk children living in underserved areas.

## Figures and Tables

**Table 1:** Characteristics of South Carolina Medicaid-enrolled children diagnosed with neonatal opioid withdrawal syndrome at birth, by rural/urban status, 2006–2014 (N = 994).

Characteristics	Rural (n = 161)	Urban (n = 833)	*p*-Value ^a^
Children			
Female, n (%)	71 (44.1)	377 (45.3)	0.79
Race and ethnicity, n (%)			0.20
White	117 (74.5)	647 (79.9)	
Black or African American	30 (19.1)	98 (12.1)	
Hispanic	2 (1.3)	12 (1.5)	
Other ^b^	7 (4.5)	43 (5.3)	
More than one race	1 (0.6)	10 (1.2)	
Gestational age, median (IQR)	38 (37–39)	38 (37–39)	0.65
Birth weight (grams), mean (SD)	2959.2 (529.1)	2950.1 (533.8)	0.85
Birth year, n (%)			0.50
2006	9 (5.6)	53 (6.3)	
2007	9 (5.6)	48 (5.8)	
2008	10 (6.2)	50 (6.0)	
2009	16 (9.9)	61 (7.3)	
2010	14 (8.7)	67 (8.0)	
2011	15 (9.3)	109 (13.1)	
2012	24 (14.9)	142 (17.1)	
2013	24 (14.9)	152 (18.3)	
2014	40 (24.8)	151 (18.1)	
Mothers			
Age, median (IQR)	27.0 (5.2)	27.6 (5.2)	0.20
Previous live births, median (IQR)	1 (1–2)	1 (0–2)	0.69
Education attained, n (%)			0.33
Less than high school graduate	59 (41.0)	270 (35.3)	
High school graduate or GED	43 (29.9)	229 (29.9)	
College credit or degree	42 (29.2)	266 (34.8)	
Kotelchuck Prenatal Care Index, n (%)			0.26
Inadequate	74 (51.4)	359 (47.2)	
Intermediate	11 (7.6)	70 (9.2)	
Adequate	16 (11.1)	130 (17.1)	
Adequate Plus	43 (29.9)	201 (26.5)	
Tobacco uses during pregnancy, n (%)	50 (34.5)	280 (36.5)	0.64

IQR = interquartile range; SD = standard deviation; GED = General Educational Development (High School Equivalency Certificate). Note: Non-normally distributed data based on the Shapiro–Wilk test statistic are presented as median (IQR). ^a^ *p*-values obtained using chi-squared tests for n (%), Student’s *t*-tests for the mean (SD), and Mann–Whitney U tests for median (IQR). ^b^ other races include Native American, Alaska Native, Asian, Native Hawaiian/Pacific Islander, and others.

**Table 2:** Mean (SD) well-child visits and immunization encounters completed during the first 3 years of life for South Carolina Medicaid-enrolled rural and urban children diagnosed with neonatal opioid withdrawal syndrome at birth, 2006–2014 (N = 994).

	Well-Child Visits			Immunization Encounters		
Age	Rural (n = 161)	Urban (n = 833)	*p*-Value ^b^	Rural (n = 161)	Urban (n = 833)	*p*-Value ^b^
Birth to 11 months	3.4 (1.7)	4.0 (2.0)	<0.001	1.6 (1.6)	2.6 (1.5)	<0.001
12 months to 23 months	1.7 (1.1)	2.1 (1.3)	<0.001	1.4 (1.3)	2.0 (1.2)	<0.001
24 months to 35 months	0.6 (0.6)	0.7 (0.6)	0.009	0.3 (0.6)	0.6 (0.7)	<0.001

SD = standard deviation. ^a^ derived using Current Procedural Terminology codes for pediatric preventive care recommended by the American Academy of Pediatrics (Supplemental Material, Table S1). ^b^ *p*-value obtained using Student’s *t*-tests for the mean (SD).

**Table 3:** Results from adjusted Poisson regression models examining associations between rural status and well-child visits and immunization encounters among South Carolina Medicaid-enrolled children with neonatal opioid withdrawal syndrome at birth, 2006–2014 (N = 994).

Age	Rural (n = 161)	Urban (n = 833)
Birth to 11 months		
Well-child visits, IRR (95% CI)	0.85 (0.77–0.93)	Ref
Well-child visit adherence, OR (95% CI) ^a^	0.81 (0.53–1.23)	Ref
Immunization visits, IRR (95% CI) ^b^	0.60 (0.52–0.69)	Ref
12 months to 23 months		
Well-child visits, IRR (95% CI)	0.80 (0.69–0.93)	Ref
Well-child visit adherence, OR (95% CI) ^a^	0.66 (0.44–0.99)	Ref
Immunization visits, IRR (95% CI) ^b^	0.60 (0.50–0.71)	Ref
24 months to 35 months		
Well-child visits, IRR (95% CI)	0.81 (0.63–1.03)	Ref
Well-child visit adherence, OR (95% CI) ^a^	0.85 (0.58–1.23)	Ref
Immunization visits, IRR (95% CI) ^b^	0.55 (0.40–0.76)	Ref

CI = Confidence interval; IRR = incidence rate ratio; OR = odds ratio. Note: Models adjusted for characteristics in Table 1. IRRs (95% CIs) and ORs (95% CIs) obtained using Poisson and logistic regression analysis, respectively. ^a^ defined as a completed well-child visit by month 1 and four additional visits by 11 months; at least two completed visits between 12 and 23 months; and at least one completed visit between 24 and 35 months based on American Academy of Pediatrics recommendations. ^b^ defined as a healthcare visit containing at least one immunization.

## Data Availability

Data are available upon request and approval from the corresponding author Farah Tahsin.

## Supplementary Materials

The following supporting information can be downloaded at https://www.mdpi.com/article/10.3390/healthcare13131539/s1, Figure S1. Cohort selection of South Carolina Medicaid-enrolled children born between 2006 and 2014 with a diagnosed neonatal opioid withdrawal syndrome at birth hospitalization; Table S1. Diagnosis and procedure codes; Table S2. STROBE statement for cohort studies; Table S3. Results from complete case analysis examining the association between rural status and well-child visits and immunizations among children with neonatal opioid withdrawal syndrome at birth, 2006–2014 (N = 994); Table S4. Results from sensitivity analyses examining associations between rural status and well-child visits and immunizations among children with neonatal opioid withdrawal syndrome at birth identified by diagnosis codes 779.5 and 760.2, 2006–2014 (N = 1407).
