# Supplementary material for: Well-Child Visits and Immunization Encounters in South Carolina Medicaid: A 3-Year Retrospective Comparison Between Rural and Urban Children with a History of Neonatal Opioid Withdrawal Syndrome, 2006–2014
Source: Healthcare (Basel). 2025 Jun 27;13(13):1539. doi: 10.3390/healthcare13131539 (PMC12250176; doi:10.3390/healthcare13131539)
Supplement: Supplementary file 1 [file healthcare-13-01539-s001.zip › healthcare-3598563-supplementary.pdf]

Supplemental Material

Figure S1. Cohort selection of South Carolina Medicaid-enrolled children born between 2006-2014 with a diagnosed neonatal abstinence syndrome at birth hospitalization.

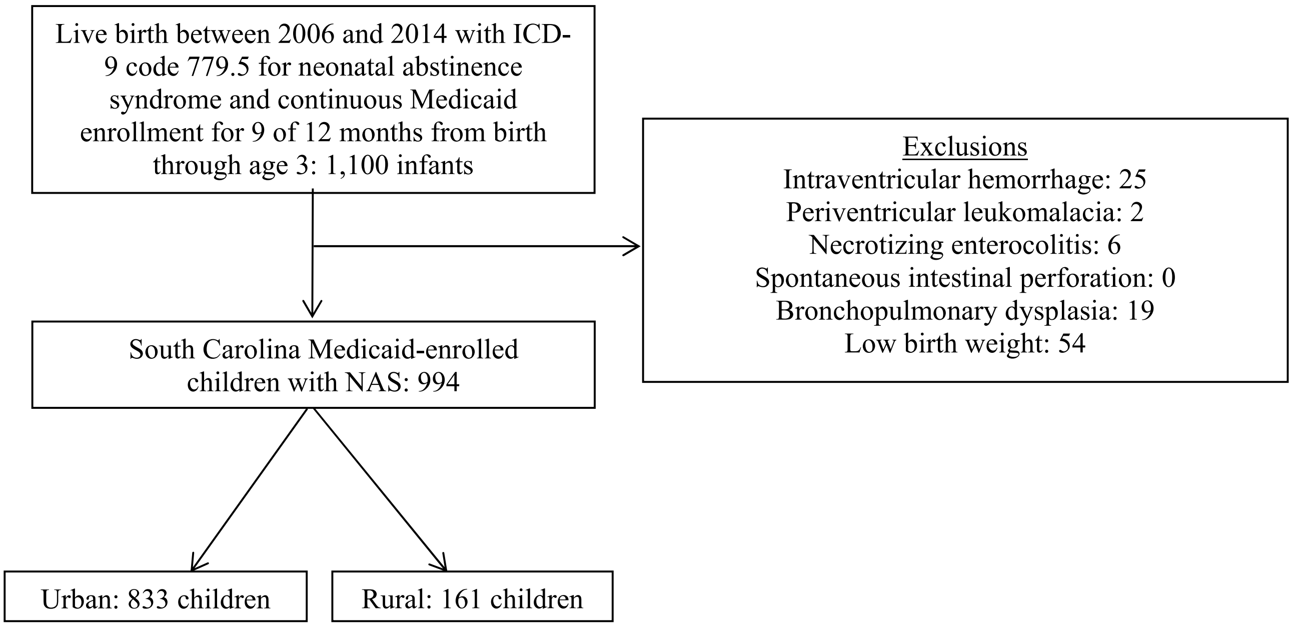

Table S1. Diagnosis and procedure codes

Exclusion criteria	ICD-9 codes
Intraventricular hemorrhage	772.1x
Periventricular leukomalacia	779.7
Necrotizing enterocolitis	777.5x
Spontaneous intestinal perforation	777.6
Bronchopulmonary dysplasia	770.7
**Outcomes**	**CPT codes**
Well-child visits (birth-3 years)	99381, 99382, 99391, 99392, 99461, G0438, G0439
Immunizations	90460, 90461, 90471, 90472, 90473, 90474, 90620, 90621, 90633, 90647, 90648, 90651, 90670, 90672, 90674, 90680, 90681, 90685, 90686, 90687, 90688, 90696, 90698, 90700, 90702, 90707, 90710, 90713, 90714, 90715, 90716, 90723, 90732, 90734, 90740, 90743, 90744, 90746, 90747, 90749, 90756

Table S2. STROBE Statement—Checklist of items that should be included in reports of ***cohort studies***

	Page No	Recommendation
**Title and abstract**	1	(*a*) Indicate the study's design with a commonly used term in the title or the abstract
		(*b*) Provide in the abstract an informative and balanced summary of what was done and what was found
**Introduction**		
Background/rationale	2	Explain the scientific background and rationale for the investigation being reported
Objectives	2	State specific objectives, including any prespecified hypotheses
**Methods**		
Study design	2	Present key elements of study design early in the paper
Setting	2-3	Describe the setting, locations, and relevant dates, including periods of recruitment, exposure, follow-up, and data collection
Participants	2-3	(*a*) Give the eligibility criteria, and the sources and methods of selection of participants. Describe methods of follow-up
		(*b*) For matched studies, give matching criteria and number of exposed and unexposed
Variables	3-4	Clearly define all outcomes, exposures, predictors, potential confounders, and effect modifiers. Give diagnostic criteria, if applicable
Data sources/ measurement	3-4	For each variable of interest, give sources of data and details of methods of assessment (measurement). Describe comparability of assessment methods if there is more than one group
Bias	3-4; 9	Describe any efforts to address potential sources of bias
Study size	3	Explain how the study size was arrived at
Quantitative variables	3-4	Explain how quantitative variables were handled in the analyses. If applicable, describe which groupings were chosen and why
Statistical methods	4	(*a*) Describe all statistical methods, including those used to control for confounding
		(*b*) Describe any methods used to examine subgroups and interactions
		(*c*) Explain how missing data were addressed
		(*d*) If applicable, explain how loss to follow-up was addressed
		(*e*) Describe any sensitivity analyses
**Results**		

Participants	3	(a) Report numbers of individuals at each stage of study—eg numbers potentially eligible, examined for eligibility, confirmed eligible, included in the study, completing follow-up, and analysed
		(b) Give reasons for non-participation at each stage
		(c) Consider use of a flow diagram
Descriptive data	4-5	(a) Give characteristics of study participants (eg demographic, clinical, social) and information on exposures and potential confounders
		(b) Indicate number of participants with missing data for each variable of interest
		(c) Summarise follow-up time (eg, average and total amount)
Outcome data	5-7	Report numbers of outcome events or summary measures over time
Main results	5-7	(*a*) Give unadjusted estimates and, if applicable, confounder-adjusted estimates and their precision (eg, 95% confidence interval). Make clear which confounders were adjusted for and why they were included
		(*b*) Report category boundaries when continuous variables were categorized
		(*c*) If relevant, consider translating estimates of relative risk into absolute risk for a meaningful time period
Other analyses	7	Report other analyses done—eg analyses of subgroups and interactions, and sensitivity analyses
**Discussion**		
Key results	7	Summarise key results with reference to study objectives
Limitations	9	Discuss limitations of the study, taking into account sources of potential bias or imprecision. Discuss both direction and magnitude of any potential bias
Interpretation	7-9	Give a cautious overall interpretation of results considering objectives, limitations, multiplicity of analyses, results from similar studies, and other relevant evidence
Generalisability	7-9	Discuss the generalisability (external validity) of the study results
**Other information**		
Funding	10	Give the source of funding and the role of the funders for the present study and, if applicable, for the original study on which the present article is based

Note: An Explanation and Elaboration article discusses each checklist item and gives methodological background and published examples of transparent reporting. The STROBE checklist is best used in conjunction with this article (freely available on the Web sites of PLoS Medicine at http://www.plosmedicine.org/, Annals of Internal Medicine at http://www.annals.org/, and Epidemiology at http://www.epidem.com/). Information on the STROBE Initiative is available at http://www.strobe-statement.org.

Table S3. Results from complete case analysis examining the association between rural status and well-child visits and immunizations among children with neonatal abstinence syndrome at birth, 2006-2014 (N=994)

**Age**	**Rural (n=161)**	**Urban (n = 833)**
Birth to 11 months		
Well-child visits, IRR (95% CI)	**0.84 (0.76-0.93)**	Ref
Well-child visit adherence, OR (95% CI)[a]	**0.56 (0.45-0.66)**	Ref
Immunization visits, IRR (95% CI)[b]	**0.60 (0.52-0.69)**	Ref
12 months to 23 months		
Well-child visits, IRR (95% CI)	**0.80 (0.69-0.93)**	Ref
Well-child visit adherence, OR (95% CI)[a]	**0.60 (0.50-0.70)**	Ref
Immunization visits, IRR (95% CI)[b]	**0.58 (0.49-0.70)**	Ref
24 months to 35 months		
Well-child visits, IRR (95% CI)	0.80 (0.63-1.03)	Ref
Well-child visit adherence, OR (95% CI)[a]	**0.54 (0.45-0.63)**	Ref
Immunization visits, IRR (95% CI)[b]	**0.54 (0.39-0.74)**	Ref

Confidence Interval; IRR = Incidence Rate Ratio; OR=Odds Ratio.
Note. Models adjusted for adjusted for characteristics in table 1. IRRs (95% CIs) and ORs (95% CIs) obtained using Poisson and logistic regression analysis, respectively. Bold font indicates statistically significant findings.
[a] Defined as a completed well-child visit by month 1 and four additional visits by 11 months; at least two completed visits between 12 and 23 months; and, at least one completed visit between 24 and 35 months based on American Academy of Pediatrics recommendations.
[b] Defined as a health care visit containing at least one immunization.

Table S4. Results from sensitivity analyses examining associations between rural status and well-child visits and immunizations among children with neonatal abstinence syndrome at birth identified by diagnosis codes 779.5 and 760.2, 2006-2014 (N=1407)

**Age**	**Rural (n=242)**	**Urban (n=1165)**
Birth to 11 months		
Well-child visits, IRR (95% CI)	**0.86 (0.80-0.93)**	Ref
Well-child visit adherence, OR (95% CI)[a]	0.85 (0.60-1.19)	Ref
Immunization visits, IRR (95% CI)[b]	**0.60 (0.54-0.68)**	Ref
12 months to 23 months		
Well-child visits, IRR (95% CI)	**0.86 (0.76-0.97)**	Ref
Well-child visit adherence, OR (95% CI)[a]	0.76 (0.56-1.03)	Ref
Immunization visits, IRR (95% CI)[b]	**0.61 (0.53-0.70)**	Ref
24 months to 35 months		
Well-child visits, IRR (95% CI)	**0.78 (0.64-0.96)**	Ref
Well-child visit adherence, OR (95% CI)[a]	0.78 (0.58-1.06)	Ref
Immunization visits, IRR (95% CI)[b]	**0.51 (0.39-0.67)**	Ref

Confidence Interval; IRR = Incidence Rate Ratio; OR=Odds Ratio.

Note. Models adjusted for adjusted for characteristics in table 1. IRRs (95% CIs) and ORs (95% CIs) obtained using Poisson and logistic regression analysis, respectively. Bold font indicates statistically significant findings.

[a] Defined as a completed well-child visit by month 1 and four additional visits by 11 months; at least two completed visits between 12 and 23 months; and, at least one completed visit between 24 and 35 months based on American Academy of Pediatrics recommendations.

[b] Defined as a health care visit containing at least one immunization.
